# Cancer-associated fibroblasts in clear cell renal cell carcinoma: functional heterogeneity, tumor microenvironment crosstalk, and therapeutic opportunities

**DOI:** 10.3389/fimmu.2025.1617968

**Published:** 2025-06-04

**Authors:** Man Wang, Yuanzhuo Zhao, Kangchun Xu, Chao Liu, Hui Zhong, You Wu, Ke Zhang, Shanzhai Wei

**Affiliations:** ^1^ Department of Nephrology, Shuyang Hospital of Traditional Chinese Medicine, Shuyang, Jiangsu, China; ^2^ The Second Clinical Medical College, Nanjing Medical University, Nanjing, Jiangsu, China; ^3^ Department of Urology, The Affiliated Suzhou Hospital of Nanjing Medical University, Suzhou Municipal Hospital, Suzhou, Jiangsu, China

**Keywords:** clear cell renal cell carcinoma (ccRCC), cancer-associated fibroblasts (CAFs), tumor microenvironment (TME), immune evasion, therapy resistance, combination immunotherapy

## Abstract

Clear cell renal cell carcinoma (ccRCC) progression heavily relies on the immunosuppressive tumor microenvironment (TME). In the ccRCC TME, the cancer-associated fibroblasts (CAFs) drive a self-perpetuating cycle of immune evasion and therapeutic resistance through diverse interactions between cells and molecules. Furthermore, heterogeneous CAFs facilitate tumor growth through metabolic reprogramming and modulate immune suppression by driving the M2 polarization of tumor-associated macrophages (TAMs) and the expansion of regulatory T cells (Tregs), which promote a multilayered immunosuppressive network. In addition, CAFs reshape the mechanical properties of extracellular matrix (ECM), hinder the infiltration of cytotoxic T lymphocytes (CTLs) and further exacerbate immune escape. Moreover, CAF-derived exosomes can confer resistance to chemoradiation therapy. Interleukin-6 (IL-6) secreted by CAFs synergizes with vascular endothelial growth factor (VEGF) to facilitate adaptive resistance to targeted therapy. Emerging therapeutic strategies—including fibroblast activation protein (FAP)-targeted CAR-T cells and transforming growth factor-β (TGF-β) inhibitors—can partially reverse this immunosuppressive property. Combination therapies employing immune checkpoint inhibitors and VEGF antagonists exhibit promising synergistic effects, although the clinical translation remains hampered by CAF heterogeneity, dual functional roles, and the lack of specific biomarkers. Future studies should integrate single-cell sequencing and spatial multi-omics techniques to comprehensively analyze the spatio-temporal dynamic heterogeneity of CAF subpopulations and develop precision treatment strategies based on molecular subtyping, aiming to break the vicious cycle of “CAF-TME-resistance” in ccRCC.

## 1 Introduction

Renal cell carcinoma (RCC) originates from the epithelial cells of the renal tubules and is a highly heterogeneous malignancy. Clear cell renal cell carcinoma (ccRCC), the predominant histological subtype of renal cancer, is principally characterized by the inactivation of the von Hippel-Lindau (VHL) tumor suppressor gene. This genetic alteration leads to constitutive activation of the hypoxia-inducible factor (HIF) pathway, which promotes metabolic reprogramming, angiogenesis, and an immunosuppressive tumor microenvironment (TME) (1).

Although targeted therapy—particularly vascular endothelial growth factor (VEGF) inhibitors—and immune checkpoint blockade have significantly improved prognosis in cancer patients, therapeutic resistance remains as a major challenge (2). This clinical dilemma highlights an urgent need of the transition from the traditional “tumor cell-centric” paradigm to a “TME-centric regulatory” conception.

The tumor microenvironment (TME) is a dynamic ecosystem composed of diverse cell types and stromal components. Among the stromal components, cancer-associated fibroblasts (CAFs) emerge as pivotal regulators that are dynamically activated through various signals involved in extracellular matrix (ECM) remodeling. Through complex molecular and cellular interactions, CAFs foster a vicious cycle of immune suppression, therapeutic resistance, and tumor progression (3).

This review systematically analyzes the multidimensional regulatory network of CAFs within the ccRCC TME, and focused on their heterogeneous origins, functional subtype, molecular mechanisms of interaction with other cells, and evolutionary potentials of CAF-targeting strategies.

## 2 Biological characteristics and functional roles of CAFs in ccRCC

### 2.1 Cellular origins and phenotypic heterogeneity of CAFs

The heterogeneity of CAFs in ccRCC is primarily attributed to their diverse cellular origins and differentiation pathways: First, resident renal fibroblasts undergo *in situ* activation into α-smooth muscle actin positive (α-SMA^+^) myofibroblasts under the influence of transforming growth factor-β (TGF-β) and platelet-derived growth factor (PDGF); Second, bone marrow-derived mesenchymal stem cells (BM-MSCs) are recruited to the TME via the CXCL12/CXCR4 axis, where they adopt CAF-like phenotypes; Third, transdifferentiation processes such as epithelial-to-mesenchymal transition (EMT) provide an additional source of CAFs (4, 5). Recent advances in single-cell RNA sequencing (scRNA-seq) have enabled a finer classification of CAF subtypes and their functional roles in ccRCC. Kieffer et al. showed in multiple solid tumor models that FAP^+^ CAFs are a key source of pro-inflammatory and immunomodulatory cytokines that impair anti-tumor immunity and confer resistance to immune checkpoint inhibitors (6). Although originally defined in pancreatic cancer, myofibroblastic CAFs (myCAFs) and inflammatory CAFs (iCAFs) exhibit conserved gene expression programs in ccRCC as well—myCAFs express TGF-β response genes and ECM components, while iCAFs secrete IL-6, LIF, and CXCL8, which amplify immunosuppression and tumor-promoting inflammation (7, 8). These findings highlight the functional specialization of CAF subsets and their relevance as therapeutic targets in ccRCC.

### 2.2 CAF-mediated tumor-promoting functions

Once activated, CAFs play an essential role in promoting tumor progression (9). Metabolically, CAFs undergo reprogramming that enhances glycolysis and facilitates the supply of metabolic intermediates to tumor cells. They secrete a variety of pro-angiogenic factors—such as CXCL12, vascular endothelial growth factor A (VEGFA), platelet-derived growth factor C (PDGFC), and osteopontin—that stimulate neovascularization. In addition, CAFs actively participate in ECM remodeling, thereby altering tissue stiffness and promoting tumor invasion and metastasis (10–12). Furthermore, CAFs also engage in direct physical and paracrine interactions with tumor cells, inducing EMT and supporting processes such as vascular mimicry (13–15). These collective actions establish a tumor-permissive microenvironment and fuel aggressive behaviors, rendering CAFs indispensable facilitators of tumor proliferation, angiogenesis, metastasis, and therapy resistance (16).

### 2.3 Signaling mechanisms driving CAF activation and differentiation

Normal fibroblasts can inhibit tumor cells proliferation and invasion, and inhibit epithelial tumors (17, 18). Therefore, the transformation of normal fibroblasts to cancer-promoting CAFs is an essential mechanism for the survival of malignant cells. The generation of CAFs results from the synergistic effect of paracrine signaling and mechanical stimulation. Once activated, CAFs create a self-reinforcing feedback loop within the TME that continuously activates these signaling pathways, driving CAF population expansion (19–21).

Paracrine signaling molecules, including transforming growth factor-β (TGF-β), interleukin-1α (IL-1α), and PDGF, play key regulatory roles in CAFs reprogramming. Specifically, the TGF-β axis has been shown to primarily drive the differentiation of myCAFs, while the IL-1α axis regulates the formation of iCAFs (22, 23). After activation of the above signaling pathways, downstream effects of CAFs reprogramming gradually emerge, such as a shift in metabolic pattern. Specifically, TGF-β-stimulated CAFs exhibit an activated oxidative stress program that provides energy substrates to cancer cells (24, 25). In addition, macrophage-derived factors and CAFs’ autocrine products together constitute additional signaling axes regulating CAFs differentiation (23, 26). This dynamic feedback network reinforces the malignant properties of CAFs, and contributes to the sustained immunosuppressive niche.

## 3 CAFs and the tumor microenvironment: a dynamic crosstalk network

### 3.1 Bidirectional interactions between CAFs and tumor cells

CAFs are essential modulators within the TME, interacting with tumor cells through various mechanisms, including tumor cell proliferation [20], regulating angiogenesis [21], construction of an immunosuppressive niche to evade immune surveillance [9], and promoting tumor formation and therapeutic resistance [22]. Through the secretion of diverse cytokines, chemokines (e.g., CXCL2), extracellular matrix proteins (e.g., collagen, laminin), and matrix metalloproteinases (MMPs), CAFs regulate immune cell recruitment, ECM remodeling, and tissue architecture (27, 28). These factors collectively facilitate cancer progression by enhancing tumor cell motility, promoting EMT, and contributing to vascular mimicry. Increasing evidence indicates that CAFs complement other components of the microenvironment to combat immune cells and regulate tumor immune microenvironment (TIME) (29, 30). CAFs coordinate the immunosuppressive TME through dynamic interactions with tumor-associated immune cells (17). Specifically, CAFs regulate immune cell-mediated anti-tumor responses through the following mechanisms: CAFs enhancing the recruitment, activation and immunosuppressive function of immunosuppressive cells (31). At the same time, the killing activity and cytokine secretion of effector immune cells (such as natural killer cells (NK cells) and cytotoxic T lymphocytes (CTLs) are inhibited, and the bidirectional regulation of immune response is realized (32, 33). Infiltrating immune cells enhance the activation state and functional activity of CAFs in both ways, thereby establishing a self-sustaining immunosuppressive feedback loop (34). CAFs induce T cell dysfunction by upregulating the expression of immune checkpoint molecules, including programmed cell death ligand 1 (PD-L1)/receptor 1 (PD-1) and cytotoxic T lymphocyte-associated protein 4 (CTLA-4)/B7, on cell surface of both TME itself and adjacent cells (35). CAFs secrete fibronectin, collagen and Matrix Metalloproteinases (MMPs), signaling pathway of Focal Adhesion Kinase (FAK) is activated to reshape the ECM (36, 37). This biomechanical remodeling further solidifies the immunosuppressive state by forming a physical barrier that prevents immune cell infiltration (37, 38). By synergistically interacting with ECM-mediated survival signals, CAFs acquire resistance against apoptosis and maintain their cancer-promoting activity (34). These tumor-supportive interactions form the basis for subsequent immunomodulatory effects within the TME.

### 3.2 Immunomodulatory Roles of CAFs in the TIME

#### 3.2.1 CAFs-TAM axis: co-amplification of innate immunosuppression

TAMs, a key component of TIME, make a significant contribution to maintaining immunosuppression (39, 40). CAFs promote the recruitment and polarization of monocytes into M2-type TAMs through secretion of factors such as IL-6, CCL2, and CXCL12. These M2-TAMs exhibit immunosuppressive phenotypes characterized by elevated PD-1 expression and reduced phagocytic activity, which impairs both innate and adaptive immune responses (41). High co-expression of CAF and M2-TAM markers (e.g., FAP and CD163) correlates with poor prognosis in ccRCC and other solid tumors (42, 43). In addition, CAFs can mediate the induction of immunosuppressive phenotypes in TAMs. For example, Gok et al. demonstrated through flow cytometry analysis that PD-1 expression is specifically elevated on M2-polarized TAMs, and CAF-mediated upregulation of PD-1 expression in TAMs significantly impaired their phagocytic capacity against tumor cells. Furthermore, this PD-1 overexpression establishes an immunosuppressive microenvironment by suppressing T cell infiltration and proliferation, while simultaneously compromising both innate and adaptive arms of anti-tumor immunity (44). This axis exemplifies the CAFs’ capacity to manipulate the innate immune compartment and primes them for interactions with adaptive immune cells.

#### 3.2.2 CAFs-Tregs axis: inhibition of adaptive immune responses

Lymphocytes, which play a crucial role in regulating adaptive immune responses, consist of different functional subgroups, including Tregs, CTLs, and Helper T (Th) cells (35, 45). There is substantial evidence of dynamic interactions between CAFs and T cell populations. The interaction between CAFs and Tregs exemplifies this immunoregulatory property (46, 47). Tregs with high Foxp3 expression have been confirmed to play a key role in inhibiting anti-tumor immunity (48, 49). Kinoshita et al. reported the spatial co-localization of Tregs and CAFs in tumor tissues (50). Clinical data further showed that co-infiltration of Foxp3^+^ Tregs and CAFs in tumor stroma was significantly associated with poor prognosis (50, 51). These findings suggest that there is a potential interaction between CAFs and Tregs. CAFs can also actively induce the phenotypic plasticity of Tregs and amplify their immunosuppressive effects (6). Chen et al. confirmed that CAF-derived TGF-β could drive the differentiation of initial T cells into CD4^+^/CD25^+^ Tregs (52). Zhao et al. reported that CAFs facilitate Treg expansion and functional activation by secreting TGF-β and promoting chemokine-driven recruitment (e.g., via CCL22 and CCL17) (53, 54). Paradoxically, Ozdemir et al. found in the pancreatic ductal adenocarcinoma (PDAC) model that myofibroblast depletion could increase the proliferation of Foxp3^+^ Tregs, thereby suppressing immune surveillance (22). This counterintuitive phenomenon suggests that CAFs-Tregs interactions have a dual, context-dependent character.

### 3.3 Mechanical remodeling of the ECM by CAFs

Fibroblasts, the primary constructors of the ECM, play a crucial role in tissue repair and homeostasis maintenance through the synthesis and remodeling of the interstitial matrix (45). CAFs play a central role in extracellular matrix remodeling, a process that not only facilitates tumor cell invasion but also modulates immune cell infiltration and accessibility. During normal wound healing, cytokines and growth factors stimulate the recruitment of fibroblasts, which respond by increasing the mechanical stress within the microenvironment. This process drives the transdifferentiation of fibroblasts into myofibroblasts, characterized by the expression of α-SMA (55). When stimulated by TGF-β1 and mechanical cues, CAFs upregulate α-SMA expression and produce fibronectin variants, such as EDA-FN. These changes facilitate the assembly of actomyosin fibers and induce persistent tissue contractility (56–58). Notably, α-SMA^+^ myofibroblasts sustain ECM contraction and drive permanent tissue remodeling, unlike smooth muscle cells, which exhibit only transient contraction properties (59). Chronic tissue contraction leads to ECM stiffening, which in turn activates TGF-β1 through tension-induced release from latent complexes. This activation further amplifies fibroblast activity via a feedforward mechanism. Additionally, actomyosin-mediated contraction of fibroblasts activates the YAP/TAZ and MRTF pathways, increasing ECM protein expression through transcriptional activation, thereby linking mechanical stress to the gene transcription of myofibroblasts (60–62). In healthy tissues, myofibroblasts return to a quiescent state or undergo apoptosis following tissue repair (63–65). However, within the tumor microenvironment, sustained signaling and mechanical tension maintain the activated state of CAFs, resulting in chronic ECM stiffness and an immunosuppressive microenvironment. These biomechanical changes impede CTL infiltration and contribute to immune evasion. Thus, ECM remodeling constitutes a mechanical barrier that acts in concert with immune suppression, further enhancing CAF-mediated resistance mechanisms.

## 4 CAF-induced immune evasion and therapeutic resistance

### 4.1 CAF-mediated construction of an immunosuppressive microenvironment

Tumors employ multiple strategies to escape immune surveillance, with CAFs involved in the TME playing a particularly crucial role in reinforcing these mechanisms (66). In ccRCC, immune evasion is primarily mediated through impaired antigen presentation, overexpression of immune checkpoints, and establishment of immunosuppressive cellular networks (67). As key architects of the immunosuppressive TME, CAFs directly impair T cell activation through secretion of TGF-β and CXCL12 (68). These mediators exert dual immunosuppressive effects: CXCL12 not only induces spatial exclusion of T cells from tumor cores but also disrupts dendritic cell maturation, while TGF-β drives the differentiation of naïve T cells into regulatory T cells (Tregs). Notably, CXCL12-mediated signaling concurrently promotes tumor angiogenesis, thereby coupling immune evasion with pro-tumorigenic processes (69). At the cellular level, ccRCC exhibits characteristic downregulation of major histocompatibility complex (MHC) molecules, severely compromising T cell-mediated tumor recognition (70). This antigen presentation defect synergizes with tumor cell upregulation of immune checkpoint molecules, which systematically inhibit T cell activation through receptor-ligand interactions (71). Immune checkpoint pathway is the core mechanism of tumor immune escape, and these checkpoints inhibit T cell activation and proliferation, which enables tumor cells to evade immunosurveillance (72). Therapeutic inhibitors targeting these checkpoints, such as anti-PD-1 antibodies and anti-CTLA-4 antibodies, are applied in treating ccRCC and other malignancies, and have shown tremendous clinical benefits (73). The presence of immunosuppressive cell populations—including TAMs, Tregs, and myeloid-derived suppressor cells (MDSCs)—further reinforces the tolerogenic environment (74). CAFs orchestrate this process through chemokine-mediated recruitment and functional reprogramming of these cell populations (75). For example, TAMs excrete cytokines and growth factors which drive tumor progression (76). On the other hand, Tregs create an immunosuppressive microenvironment by inhibiting T cell activation and secreting TGF-β and interleukin-10 (IL-10), which promote tumor evasion of immunosurveillance (77). Importantly, CAFs do not only coexist with but also actively coordinate these immunosuppressive pathways, and these roles could establish an immunological framework that facilitates subsequent therapy resistance development.

### 4.2 Molecular mechanisms of CAF-driven therapy resistance

Theatment resistance is one of the reasons for treatment failure, and tumor recurrence in cancer patients. CAFs contribute to this resistance through both direct and indirect mechanisms, including the release of exosomes, paracrine signaling, and metabolic modulation. One of the most well-characterized mechanisms involves exosomes derived from CAFs, which are enriched in non-coding RNAs such as miR-590-3p. These exosomes are taken up by tumor cells, where they activate the PI3K/AKT signaling pathway, thereby inhibiting apoptosis and promoting radioresistance (78, 79). In colorectal cancer (CRC), similar exosomal cargo has been shown to suppress cleaved caspase-3 activity, a key mediator of programmed cell death, suggesting a conserved mechanism across tumor types (80). Beyond exosomal communication, CAFs secrete cytokines that modulate tumor cell plasticity and resistance. For example, IL-6, frequently overexpressed by CAFs, synergizes with VEGF to support tumor angiogenesis and adaptive resistance to VEGF-targeted therapies (81). CAFs also produce stromal-derived factor-1 (SDF-1/CXCL12), which has been implicated in tumor cell survival and evasion of anti-angiogenic treatments (82). Taken together, these findings underscore the centrality of CAFs in facilitating immune evasion and treatment failure. Addressing CAF-driven resistance requires a comprehensive understanding of their signaling networks, which will guide the development of combination therapies capable of disrupting this malignant crosstalk.

## 5 Therapeutic strategies targeting CAF–TME interactions in ccRCC

**Table 1** summarizes representative ongoing clinical studies evaluating CAF-related biomarkers across solid tumors and sets the stage for subsequent therapeutic strategies (Data source: ClinicalTrials.gov: https://beta.clinicaltrials.gov/ provided by the U.S. National Library of Medicine).

**Table 1:** Overview of CAF-related biomarker applications in solid tumor clinical studies.

Indication	Phase	Context	NCT IDs
FAP			
Breast cancer	Diagnostic	Visualize fibroblast activation protein in breast tumor stroma using ^68^Ga-FAPI PET imaging.	NCT05574907
Gastrointestinal malignancies	II	Quantify FAP-expressing cells via ^18^F-FAPI-74 PET to assess tracer sensitivity in GI tumors.	NCT05641896
Various solid tumors (low FDG uptake)	Diagnostic	Compare diagnostic performance of ^18^F-FAPI vs. ^18^F-FDG PET/CT in lesions with minimal FDG avidity.	NCT05034146, NCT05485792
Advanced solid tumors	I	Determine safety and tolerability of OMTX7 in combination with pembrolizumab in advanced malignancies.	NCT05547321
Meflin			
Pancreatic adenocarcinoma	I/II	Assess dose-limiting toxicity and optimize dosing of Am80 plus gemcitabine and nab-paclitaxel.	NCT05064618
FGFR/PDGFR/VEGFR			
NSCLC	I/II	Evaluate tolerability and preliminary efficacy of nintedanib combined with PD-1 and CTLA-4 inhibitors.	NCT03377023
Advanced solid tumors	Ib	Explore anti-angiogenic synergy with PD-1 blockade on tumor growth inhibition.	NCT02856425
MCT-4/Caveolin-1			
Breast carcinoma	I (completed)	Investigate metabolic modulation of breast cancer cells following N-acetylcysteine administration.	NCT01878695
Pancreatic carcinoma	Preclinical	Establish patient-derived organoid platforms to predict drug response targeting MCT-4 and caveolin-1.	NCT05571956, NCT05196334

### 5.1 Molecular targeting of CAFs and their signaling pathways

Given the multifaceted roles of CAFs in tumor progression, immune evasion, and therapeutic resistance, direct targeting of these cells has emerged as a rational therapeutic strategy. These approaches include selective depletion, functional inhibition, or reprogramming of CAFs to attenuate their tumor-promoting effects. A primary strategy involves targeting surface biomarkers specific to CAFs (15). FAP, a serine protease overexpressed on CAFs in multiple tumor types, has become a leading candidate. A number of studies have showed FAP-directed chimeric antigen receptor (CAR) T cells demonstrated potent antitumor effects in preclinical models (83–85). However, the widespread expression of FAP in non-malignant tissues poses a significant challenge for clinical translation, particularly due to potential stromal toxicity. In addition to surface markers, CAF-derived cytokines and signaling pathways have been explored as therapeutic targets. The blockade of TGF-β signaling, a master regulatory axis governing CAF activation, using selective inhibitors like Galunisertib has demonstrated therapeutic efficacy in mitigating CAF-driven ECM fibrogenesis while concomitantly enhancing CD8^+^ T cell immunosurveillance (86). Moreover, CAFs influence the TME through other signaling circuits, such as IL-6/STAT3 and Hedgehog pathway, which have become attractive drug targets. Emerging compounds that inhibit these axes may indirectly mitigate CAF activity and sensitize tumors to conventional and immune therapies [80]. Epigenetic regulators, including histone deacetylase (HDAC) and Smoothened (SMO) inhibitors, also influence CAF reprogramming and stromal remodeling. These agents are under investigation for their dual activity on both tumor cells and the surrounding microenvironment. Thus, CAF-directed interventions hold promise as standalone or adjunctive therapies.

### 5.2 Combination strategies with immunotherapy to overcome resistance

As summarized in **Table 2**, several phase II/III trials—including CheckMate 214, KEYNOTE-426, and CLEAR—have established ICI-based regimens as the cornerstone of first-line therapy for advanced ccRCC. The complex crosstalk between CAFs and immune components in the TME provides a compelling rationale for combining CAF-targeting approaches with immunotherapy. Immune checkpoint inhibitors (ICIs), including those targeting PD-1/PD-L1 and CTLA-4 pathways, have revolutionized treatment paradigms for advanced ccRCC. However, their efficacy remains limited in CAF-rich tumors due to impaired T cell infiltration, sustained immune suppression, and stromal resistance mechanisms (87, 88). A clinical trial of combination therapy based on an immune checkpoint inhibitor recently confirms, combination therapy is significantly better than monotherapy in an untreated patient with metastatic ccRCC (89). A clinical trial in melanoma patients compared the efficacy of anti-CTLA4 antibody (ipilimumab) alone with the combined gp100 peptide vaccine, showing that the first two regimen significantly improved overall survival (86, 90). The CheckMate 214 study highlighted the fact that nivolumab combined with ipilimumab achieved higher objective response rates and longer overall survival compared to sunitinib in patients with untreated metastatic ccRCC. However, these effects are still compromised by the suppressive stromal environment, which attenuates immune infiltration and facilitates adaptive resistance (91). To address this challenge, combination regimens integrating CAF-targeted agents with ICIs are being actively explored. TGF-β inhibitors, such as Galunisertib and FAK inhibitors, have been shown in preclinical models to modulate the fibrotic and immunosuppressive properties of the stroma, thereby improving immune cell infiltration and sensitizing tumors to immunotherapy. These strategies not only enhance the efficacy of ICIs but also reverse the CAF-driven immune exclusion phenotype (92). Interestingly, the depletion of CAFs is not always beneficial. Some studies suggest that total ablation may lead to compensatory immunosuppressive mechanisms, such as increased Treg infiltration. Therefore, the focus has shifted toward functional reprogramming or partial inhibition to normalize CAF behavior without eliminating their structural roles. Adoptive cell therapies such as CAR-T cells targeting CAF-specific antigens like FAP have also shown potential in reversing immune resistance in solid tumors. Although promising, these approaches require further optimization to mitigate off-target toxicities and ensure safe, durable responses in patients (93). The overall response rate, overall survival rate, and objective response rate (ORR) in the nivolumab plus ipilimumab group were better. Analysis of patients after recovery showed that compared with Sunitinib, the combination regimen of nivolumab could significantly improve patients’ health status (94, 95). Moreover, the addition of CAF-targeting agents has the potential to modulate other immune checkpoints beyond PD-1 and CTLA-4, enhancing the breadth and depth of immune activation. Combination strategies are being tested in ongoing clinical trials and represent a promising path to maximize the benefit of immunotherapy in CAF-rich tumors (91, 96). Together, these approaches provide a foundation for rational combination therapies that address not only tumor-intrinsic immune escape but also the stromal impediments orchestrated by CAFs.

**Table 2:** Key clinical trials of immune checkpoint inhibitor monotherapy and combination regimens in renal cell carcinoma.

NCT Number	Trial	Phase	Line	Patients (n)	Regimen	Comparator	mOS (mo) [HR (95% CI), p]	mPFS (mo) [HR (95% CI), p]	ORR (%) [95% CI]
NCT02231749	CheckMate 214	III	First line	1,096 pts	Nivolumab + Ipilimumab	Sunitinib	47.0 v 26.6 [0.68 (0.58–0.81), p<0.0001]	11.6 v 8.4 [0.82 (0.64–1.05), p=0.03]	42 v 27 (37–47 v 22–31)
NCT02684006	JAVELIN Renal 101	III	First line	886 pts	Avelumab + Axitinib	Sunitinib	NE v NE	13.3 v 8.0 [0.69 (0.57–0.83), p=0.0001]	53 v 27 (48–57 v 23–32)
NCT02853331	KEYNOTE-426	III	First line	861 pts	Pembrolizumab + Axitinib	Sunitinib	NR v 35.7 [0.53 (0.38–0.74), p<0.0001]	15.4 v 11.1 [0.71 (0.60–0.84), p<0.0001]	59 v 36 (55–64 v 31–40)
NCT02420821	IMmotion 151	III	First line	915 pts	Atezolizumab + Bevacizumab	Sunitinib	36.1 v 35.3 [0.91 (0.76–1.08), p=0.27]	9.6 v 8.3 [0.88 (0.74–1.04), p=0.12]	37 v 33 (32–41 v 29–38)
NCT02811861	CLEAR	III	First line	NR	Lenvatinib + Everolimus ± Pembrolizumab	Sunitinib	NR v NR [0.66 (0.49–0.88), p=0.005]	23.9 v 9.2 [0.39 (0.32–0.49), p<0.001]	71 v 36 (66–76 v 48–59)
NCT03141177	CheckMate 9ER	III	First line	NR	Nivolumab + Cabozantinib	Sunitinib	NR v NR [0.60 (0.40–0.89), p=0.001]	16.6 v 8.3 [0.51 (0.41–0.64), p<0.0001]	56 v 27 (50–61 v 22–32)
—	CheckMate 025	III	Second line	821 pts	Nivolumab	Everolimus	25.0 v 19.6	4.6 v 4.4	25 v 5
NCT01472081	CheckMate 016	I	Second line	47 v 47	Nivo 3 + Ipi 1	Nivo 1 + Ipi 3 mg/kg	NR v 32.6 [0.80 (0.62–1.03), p=0.0392]	7.7 v 9.4 (3.7–14.3 v 5.6–18.6)	40 v 40 (26–56)
NCT02501096	KEYNOTE-146	Ib/II	Second line	145 pts	Lenvatinib + Pembrolizumab	—	32.2 (29.8–55.8)	14.1 (11.6–18.4)	63 (55–71)
—	PROSPER RCC	III	Adjuvant	NR	Nivolumab	Observation	–	–	–
—	CheckMate 914	III	Adjuvant	NR	Nivolumab + Ipilimumab	Placebo	–	–	–
—	KEYNOTE-564	III	Adjuvant	NR	Pembrolizumab	Placebo	–	–	–
—	IMmotion 010	III	Adjuvant	NR	Atezolizumab	Placebo	–	–	–

### 5.3 Integrative approaches combining anti-VEGF and immunotherapies

**Figure 1:**
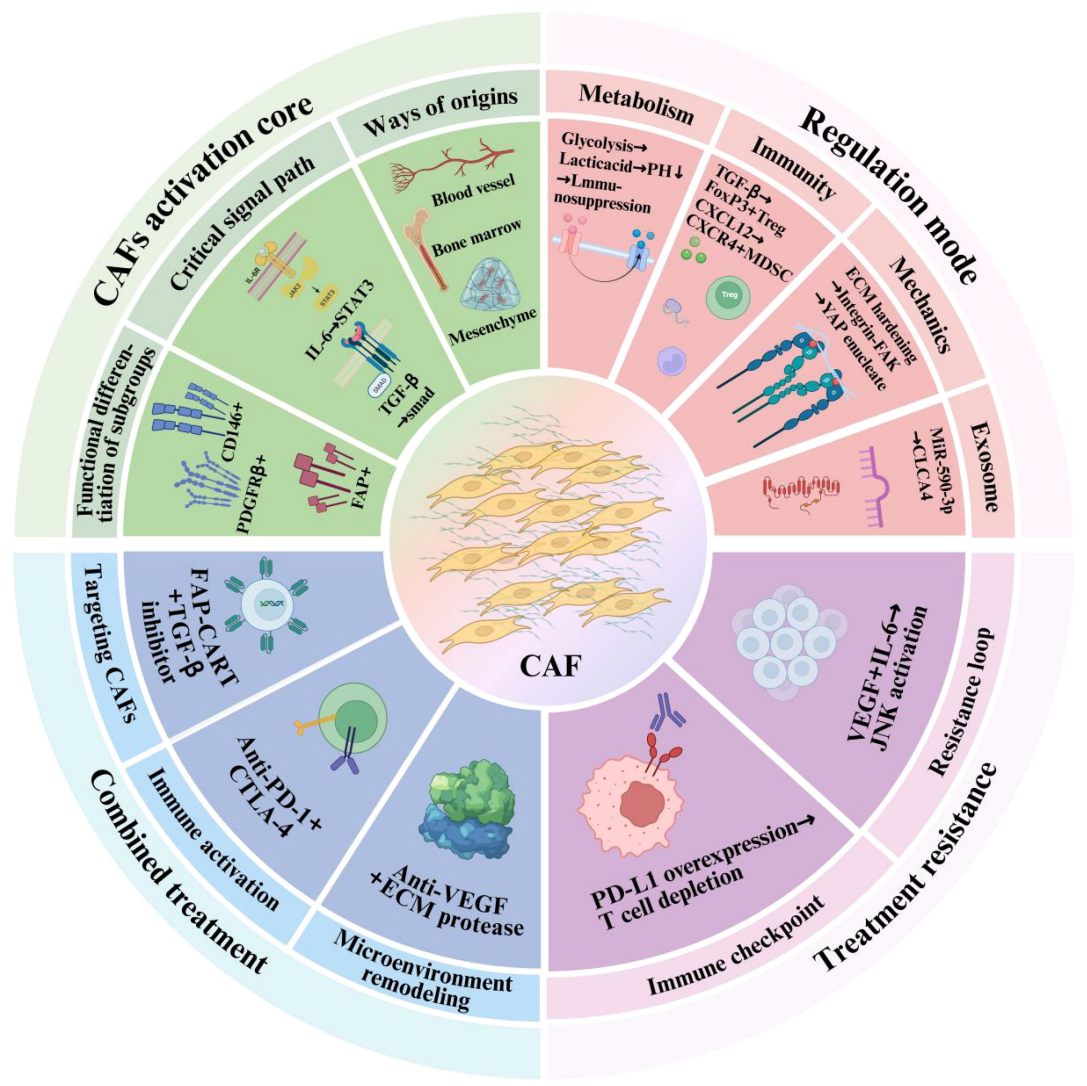
Functional frameworks of cancer-associated fibroblasts (CAFs) in tumor progression and therapy. The inner green sector highlights CAF activation, including their cellular origins (e.g., mesenchymal, bone marrow, endothelial), major signaling pathways (e.g., IL-6/STAT3, TGF-β), and functional subgroups (e.g., FAP^+^, PDGFRβ^+^). The pink sector shows the regulatory mechanisms by which CAFs influence the TME, including metabolic reprogramming, immunosuppression, mechanical remodeling, and exosome-mediated communication. The outer blue and purple regions demonstrate the involvement of CAFs in treatment resistance and combination therapy strategies, including immune checkpoint blockade, anti-VEGF therapy, and CAF-targeting interventions.

Standard-of-care treatment for metastatic ccRCC has traditionally relied on VEGF-targeted tyrosine kinase inhibitors (TKIs) (97). These agents inhibit angiogenesis, which is critical in ccRCC due to its highly vascular nature. However, monotherapies targeting VEGF pathways often lead to adaptive resistance, limited response duration, and enhanced tumor aggressiveness, partly due to immune evasion and compensatory stromal signaling. In addition to stimulating angiogenesis, VEGF not only promotes neovascularization but also contributes to immune suppression by enhancing the infiltration and function of immunosuppressive cell subsets such as Tregs and MDSCs, as well as by inhibiting dendritic cell maturation (98, 99). Consequently, combining VEGF inhibition with immunotherapies has emerged as a promising strategy to both normalize the tumor vasculature and relieve immunosuppressive constraints within the TME. Multiple clinical trials have investigated these integrative approaches. For instance, the combination of bevacizumab (anti-VEGF antibody) with interferon-alpha showed survival benefits in metastatic RCC patients. More recent data from trials evaluating ICIs plus VEGF-TKIs—such as pembrolizumab with axitinib or nivolumab with cabozantinib—demonstrated significantly improved objective response rates and progression-free survival compared to monotherapy (96, 100). From a mechanistic perspective, anti-VEGF therapy reduces vascular permeability and interstitial pressure, thereby enhancing immune cell trafficking into tumors. When combined with ICIs, this dual action fosters a reactivation of anti-tumor immunity. Importantly, VEGF blockade also indirectly modulates CAF behavior by altering paracrine signaling and ECM remodeling dynamics (101). Nevertheless, treatment optimization requires deeper insight into the temporal dynamics of VEGF-immune-CAF interactions and the identification of biomarkers predictive of response. Integrating spatial multi-omics and longitudinal immune profiling could facilitate precision stratification and enhance clinical outcomes (102). In summary, the combination therapy of anti-VEGF agents and ICIs represents a synergistic modality that addresses both vascular and immunological components of the TME. When further integrated with CAF-targeting strategies, such combinations hold the potential to overcome multidimensional resistance and transform the treatment landscape of advanced ccRCC. An integrative schematic overview of CAF-related signaling, regulation, and therapeutic implications is presented in **Figure 1**.

## 6 Conclusions and prospects

TME plays a pivotal role in the development, progression, and therapeutic response of ccRCC. Among these constituents within TME, CAFs have emerged as central orchestrators of immune evasion, treatment resistance, and stromal remodeling. Recent advances in single-cell sequencing and spatial transcriptomics have revealed the phenotypic and functional heterogeneity of CAFs and enabled the identification of distinct subsets with angiogenic, immunosuppressive, and ECM-remodeling properties. Therapeutic strategies targeting CAFs are advancing rapidly. Agents aimed at CAF-specific surface markers (e.g., FAP, PDGFRβ), secreted factors (e.g., TGF-β, IL-6), and key regulatory pathways (e.g., Hedgehog, STAT3, WNT) have progressed preclinical or clinical development. Functional reprogramming of CAFs, rather than complete depletion, appears to be a safer and potentially more effective approach, given their context-dependent pro- and anti-tumor functions. Furthermore, combining CAF-targeted interventions with ICIs and anti-angiogenic agents has demonstrated promising synergistic effects. These integrative strategies address not only tumor-intrinsic mechanisms but also the stromal and immune components of resistance. However, their clinical translation faces challenges from CAF heterogeneity, dynamic plasticity, off-target effects, and the lack of validated biomarkers to predict therapeutic response. To overcome these barriers, future studies should focus on the integration of single-cell and spatial multi-omics profiling to map the spatial and temporal evolution of CAF subtypes and their interactions with other TME elements. Identifying molecular signatures associated with response or resistance is crucial for guiding precision treatment. Ultimately, disrupting the vicious cycle between CAFs, the TME, and therapy resistance holds great promise for improving outcomes in patients with advanced ccRCC. As our understanding deepens, CAFs are likely to shift from therapeutic challenge to therapeutic opportunity in the evolving landscape of renal cancer treatment.
